# A systematic review of Hepatitis C virus treatment uptake among people who inject drugs in the European Region

**DOI:** 10.1186/1471-2334-14-S6-S16

**Published:** 2014-09-19

**Authors:** Jeffrey V Lazarus, Ida Sperle, Mojca Maticic, Lucas Wiessing

**Affiliations:** 1 CHIP, Centre for Health and Infectious Disease Research and WHO Collaborating Centre on HIV and Viral Hepatitis, Rigshospitalet, University of Copenhagen, Copenhagen, Denmark; 2 Clinic for Infectious Diseases and Febrile Illnesses, University Medical Centre Ljubljana, Ljubljana, Slovenia; 3 European Monitoring Centre for Drugs and Drug Addiction (EMCDDA), Lisbon, Portugal

## Abstract

**Background:**

Fifteen million adults in the World Health Organization European Region are estimated to have active hepatitis C infection. Intravenous drug use is a major hepatitis C transmission route in this region, and people who inject drugs (PWID) constitute a high-risk and high-prevalence population. A systematic review was conducted to assess levels of hepatitis C treatment uptake among PWID in Europe.

**Methods:**

Searches in MEDLINE and EMBASE were carried out for articles in any language published between 1 January 2000 and 31 December 2012. Articles were included in the review if they presented original research findings about hepatitis C treatment uptake levels among people who reported injecting drugs currently or formerly, as well as those who reported using drugs currently or formerly (mode of consumption not specified). Treatment uptake data were extracted if uptake was measurable in relation to the number of patients who either: (a) tested HCV antibody-positive; (b) tested positive for HCV-RNA; or (c) tested positive for HCV-RNA and met additional treatment criteria.

**Results:**

Twenty-five articles from 12 countries were included in the review. Among groups of drug-using study participants who were hepatitis C antibody-positive, the median treatment uptake level was 17%, and among those who were hepatitis C RNA-positive, the median was 30%. In the 11 studies reporting specifically on treatment uptake among current and former injecting drug users, hepatitis C RNA-positive study populations had a median treatment uptake level of 32%. Only one study reported on treatment uptake for current drug users.

**Conclusions:**

This systematic review indicates that hepatitis C treatment uptake is relatively low among drug users in several European countries, and also points to considerable knowledge gaps regarding treatment uptake levels in this population. There was large variability in treatment uptake levels, suggesting that there may be major differences between and within countries in relation to treatment availability, drug-using populations in need of treatment, and the existence of integrated health care services targeting drug users. Stronger national hepatitis C treatment policies are needed, along with efforts to increase knowledge and reduce misconceptions among physicians regarding the feasibility and importance of treating drug users who have hepatitis C.

## Introduction

An estimated 185 million people worldwide have acquired the hepatitis C virus (HCV) [1], many of them without being aware of their infection. Chronic disease can be expected to occur in 55% to 85% of untreated cases, and potential long-term outcomes for chronically infected people include liver cirrhosis, liver failure and hepatocellular carcinoma [2]. A 2006 assessment of the global burden of disease from hepatitis B and hepatitis C put annual HCV-related mortality at 366,000 [3], while more recent research yielded an estimate of 499,000 deaths due to HCV in 2010 [4].

In the World Health Organization (WHO) European Region, 15 million adults are estimated to have active HCV infection as defined by the presence of HCV-RNA. This translates into a regional adult prevalence rate of 2.0% [5]. While the limitations of the available data invite some uncertainty about the magnitude of the HCV epidemic in the region overall, numerous studies provide evidence of high HCV antibody levels (indicating either current or previous infection) in specific countries and subnational regions. For example, a 2013 review article identified reports of HCV antibody prevalence levels in the general population ranging from 0.1% to 22% at the national and subnational level in 13 European countries. The article observed that prevalence was lower in northwestern European countries and higher in the countries of the south and southeast [6].

Injecting drug use is a major driver of the HCV epidemic in Europe. According to the 2013 *European Drug Report: Trends and Developments*, injecting drug use accounted for 58% of all HCV diagnoses across 18 European countries for which 2010/2011 data were available [7]. An analysis of HCV-RNA prevalence data from the WHO European Region concluded that two million of the region’s 15 million adults with HCV-RNA are people who inject drugs (PWID) [5]. While wide-ranging HCV antibody prevalence levels have been found across different PWID cohorts, overall prevalence in this population appears to be much higher than in the general population. According to a 2013 systematic review, HCV antibody prevalence among PWID in 29 European countries ranged from 5% to 90% [6]. The 2013 *European Drug Report* noted that in eight of 12 countries with HCV antibody data from national samples of PWID, prevalence exceeded 40% [7].

Effective and safe HCV treatment can be used in the majority of infected patients and would greatly reduce the associated morbidity and mortality. Several studies have shown that HCV treatment outcomes in PWID are comparable to those in patients with no history of drug use [8,9]. Additionally, treatment also helps to prevent transmission by eliminating the potential source of infection [10]. Nevertheless, current treatment uptake overall is low, and treatment rates appear to be lowest among the most affected at-risk group: PWID [11]. Barriers to HCV treatment are most likely to be present on different levels including the patient, provider and system levels [11]. These barriers may include a lack of knowledge, a lack of financial resources and a fear of side-effects among patients, as well as concerns of adherence and the risk of re-infection at the provider level [11]. Another barrier is the setting itself, which needs to be suitable for this group and able to adequately handle different needs in this vulnerable population [12] as well as address associated stigma [13].

The purpose of this article is to systematically review the evidence on hepatitis C virus treatment uptake among PWID in the WHO European Region.

## Methods

**Figure 1:**
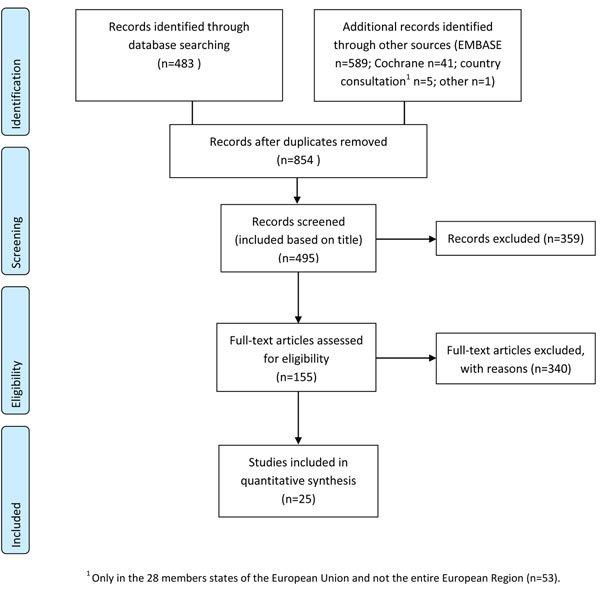
Flow diagram of study selection

We performed a systematic review of literature on HCV treatment uptake among PWID in the WHO European Region (Figure 1). Searches were carried out in MEDLINE and EMBASE for articles in any language published between 1 January 2000 and 31 December 2012. A sensitive search string (available upon request) was developed with keywords covering hepatitis C virus, substance abuse, geographic scope and access to treatment. Additional articles, including grey literature articles, were located through e-mail consultations with a network of experts representing 27 European Union countries. The protocol was made consistent with the PRISMA criteria as described elsewhere [14,15]. This study draws upon the protocol of a similar systematic review on treatment uptake among PWID [15], but important differences in inclusion and exclusion criteria and reporting of the results explains the differences in the findings.

Two researchers independently screened search results for relevance on the basis of titles and abstracts, then compared their findings. When there was uncertainty about the relevance of an item, it was retained for further consideration. Next, two researchers independently evaluated all potentially relevant articles on the basis of the full article text using predetermined inclusion and exclusion criteria (Box 1). Additional articles were identified and screened by examining the reference lists of articles found to be eligible for inclusion. The final set of articles underwent data extraction. Native speakers provided assistance with screening and data extraction for non-English articles, and online translation services (Google Translate and BabelFish) were utilised as well.

### Box 1. Inclusion and exclusion criteria for articles

#### Inclusion criteria

• Original research findings about treatment uptake levels in HCV patient cohorts (not articles reporting on modelling findings or systematic reviews).

• Study participants who reported injecting drugs currently or formerly as well as those who reported using drugs currently or formerly (mode of consumption not specified).

• Treatment uptake level measurable in relation to the number of patients who either: (a) tested HCV antibody-positive; (b) tested positive for HCV-RNA; or (c) tested positive for HCV-RNA and met additional treatment criteria.

• Data collected after 1990.

• Unselected study population.

#### Exclusion criteria

• Study cohort comprised solely of people who reported non-injecting drug use.

• Study findings based on modelling rather than actual patient data.

• Use of hepatitis C treatment determined by patient self-report.

• Confusing, ambiguous or self-contradictory study findings.

The following primary and secondary outcomes of interest guided data extraction. The primary outcome of interest was the proportion of drug users initiating HCV treatment from among all drug users who were candidates for HCV treatment; this was regarded as the treatment uptake level. Drug users generally rather than PWID were deemed the population of interest because it was noted that few studies appeared to clearly differentiate among these two populations when reporting treatment uptake levels. We defined candidates for HCV treatment as those patients who either: (a) tested HCV antibody-positive; (b) tested positive for HCV-RNA; or (c) tested positive for HCV-RNA and met additional treatment criteria specified in the study protocol. Secondary outcomes of interest were treatment uptake levels for specific types of patient cohorts (e.g., current drug users); levels of sustained viral response (SVR) in treated cohorts; and findings about the age and sex of patients obtaining treatment.

## Results

### Studies included in review

Table 1 provides characteristics of the 25 studies included in the review. The studies were carried out in 12 countries. The country with the largest number of studies was France, with five. Germany, Ireland, Spain and the United Kingdom all had three studies. (One of the three German studies also included study sites in Austria.)

**Table 1:** Characteristics of included studies

Author, year	Country	Study design	Study population
***Study setting: drug treatment clinic***			

Backmund et al, 2001	Germany	Prospective cohort study	100 drug users who were diagnosed HCV-RNA positive while receiving inpatient detoxification treatment. HCV treatment exclusion criteria included severe depression and HIV-positive diagnosis.

Ebner et al, 2009	Austria	Randomised controlled study	75 HCV-RNA positive drug users diagnosed with opioid dependence and receiving addiction treatment services. Patients were not eligible for HCV treatment unless they were undergoing drug maintenance therapy and/or had not used illicit substances for ≥ 6 months.

Grando-Lemaire et al, 2002	France	Prospective cohort study	225 drug users found to be HCV antibody-positive while they were receiving services at an addiction outpatient unit. Patients were further screened for treatment eligibility via HCV-RNA testing and liver biopsy.

Guadagnino et al, 2007	Italy	Prospective cohort study	169 HCV-RNA positive PWID identified at 11 drug dependency units. These patients were referred to collaborating infectious diseases clinical centres for evaluation regarding their suitability for HCV treatment, then the drug dependency units supervised treatment.

McCormick et al, 2008	Ireland	Prospective cohort study	13 drug treatment clinic patients who were diagnosed HCV-RNA positive and met study inclusion criteria. These included being stable on methadone; having a six-month record of abstinence from intravenous drug use; and having HCV genotype 2 or 3.

Moussalli et al, 2010	France	Cross-sectional study	337 patients at a drug users’ addiction centre: 113 diagnosed HCV-RNA positive before an HCV intervention was introduced, and 224 diagnosed HCV-RNA positive after the introduction of the intervention, which included an on-site multidisciplinary health care team.

Schulte et al, 2010	Germany	Prospective cohort study	301 HCV antibody-positive people receiving heroin maintenance at five drug treatment centres. The hepatitis C treatment study was nested within a heroin maintenance study.

van der Veen, 2009	Netherlands, the	Retrospective cohort study	Patient files examined for 115 HCV-RNA positive PWID at a drug treatment clinic.

Wilkinson et al, 2008	United Kingdom	Retrospective cohort study	441 HCV-RNA positive drug users identified by offering HCV testing to all patients at a drug treatment clinic and referringtreatment candidates to a monthly hepatology outreach clinic within the drug treatment unit.

Witteck et al, 2011	Switzerland	Cross-sectional study	193 HCV-RNA positive patients identified in opioid substitution programmes in two Swiss cities.

***Study setting: hepatitis clinic***			

Cournot et al, 2004	France	Retrospective cohort study	Data for 225 HCV antibody-positive PWID including 41 current PWID analysed as part of a study of a larger cohort of 435 people attending inpatient and outpatient clinics at a hospital hepato-gastroenterology unit.

Crespo et al, 2001	Spain	Prospective cohort study	416 HCV antibody-positive PWID were among 1269 people referred for therapeutic evaluation at a university hospital-based hepatitis clinic.

Gazdag et al, 2010	Hungary	Retrospective cohort study	83 HCV-RNA positive PWID at a hepatology outpatient clinic.

Jowett et al, 2000	United Kingdom	Retrospective cohort study	253 HCV antibody-positive patients who attended a regional (referral) liver unit and who had reported injecting drug use as their main risk factor for HCV.

Kieran et al, 2011	Ireland	Retrospective cohort study	Subset of 327 HCV-RNA positive people reporting injecting drug use as their HCV risk factor at a clinic for the integrated management of HIV and HCV.

***Study setting: other/unknown***			

Broers et al, 2005	Switzerland	Prospective cohort study	22 PWID included in a cohort of 27 patients offered treatment upon being diagnosed with acute or subacute hepatitis C.

Cullen et al, 2007	Ireland	Retrospective cohort study	104 HCV antibody-positive drug users who were among a cohort of 196 drug users prescribed methadone maintenance by general practitioners.

Defossez et al, 2008	France	Cross-sectional study	HCV antibody-positive people who reported drug use as a presumed mode of infection in cross-sectional studies of patients newly diagnosed with HCV in 1997, 2000 and 2003. (Total numbers of newly diagnosed patients were 69, 58 and 96 respectively.)

Hernandez et al, 2009	Spain	Retrospective cohort study	Four HCV-RNA positive PWID in a hospital-based setting.

Jack et al, 2009	United Kingdom	Prospective cohort study	174 HCV antibody-positive drug users engaged in drug treatment services in a primary care environment.

Kristensen et al, 2009	Norway	Prospective cohort study	Heroin-dependent patients in medical rehabilitation.

Lindenburg et al, 2011	Netherlands, the	Prospective cohort study	277 HCV antibody-positive drug users referred to a hepatitis C treatment project for drug users.

Perez-Alvarez et al, 2012	Spain	Retrospective cohort study	27 PWID within a cohort of 131 patients with acute HCV at 18 Spanish hospitals.

Perut et al, 2009	France	Retrospective cohort study	137 currently opioid-dependent people included in a cohort of HCV-RNA positive inpatients or outpatients at a Paris hospital.

Reiberger et al, 2011	Austria and Germany	Retrospective cohort study	Study of HIV/HCV co-infected patients in 14 specialised clinical centres included 637 people who reported injecting drug use as HCV transmission route.

The 25 studies included one randomised controlled study [16], three cross-sectional studies [17-19], 11 retrospective cohort studies [20-30] and 10 prospective cohort studies [31-40]. Ten of the studies took place at drug treatment clinics, five at hepatitis clinics, and ten in other types of settings.

### Overall treatment uptake levels and sustained viral response

Treatment uptake levels were assessed for this review using three different metrics. Some studies reported treatment uptake in terms of more than one metric.

The *HCV antibody-positive treatment uptake level* reflects the number of study participants who received treatment as a proportion of the number of study participants known to have antibodies for hepatitis C. Ten studies [19,22,24,26,32,35,36,38-40] reported treatment uptake in relation to HCV antibody-positive status (Table 2). Among groups of study participants who were HCV antibody-positive, treatment uptake ranged from 3% to 64% (median: 17%).

**Table 2:** Treatment uptake as defined by percentage of hepatitis C antibody-positive study participants who received treatment

Author, year	% treated (# treated/# of treatment candidates)	SVR (%) (# with sustained viral response/# treated)
Cournot et al, 2004	36% (81/225)	Intent-to-treat: 26% (26/99)^1^

Crespo et al, 2001	64% (268/416)	NA

Cullen et al, 2007	3% (3/104)	NA

Defossez et al, 2008	16% (13/84)^2^	NA

Grando-Lemaire et al, 2002	12% (27/225)	Intent-to-treat: 19% (5/27) As-treated: 33% (5/15)

Jack et al, 2009	17% (30/174)	Intent-to-treat: 62% (13/21) As-treated: 77% (13/17)

Jowett et al, 2000	20% (50/253)	Intent-to-treat: 36% (18/50)

Kristensen et al, 2009	4% (6/160)	NA

Lindenburg et al, 2011	21% (58/277)	Intent-to-treat: 65% (37/57) As-treated: 77% (37/48)

Schulte et al, 2010	9% (26/301)	Intent-to-treat: 69% (18/26) As-treated: 86% (18/21)

1. Calculated according to the number of treatment outcomes (N=99) rather than the number of patients (N=81)

2. Study population represents study cohorts from a cross-sectional survey carried out at three time points: 1997 (N=35), 2000 (N=19) and 2003 (N=30).

The *HCV RNA-positive treatment uptake level* reflects the number of study participants who received treatment as a proportion of the number of study participants known to be hepatitis C RNA-positive. Twenty-one studies [16-18,20-34,37,38,40] yielded 22 datasets with information about HCV RNA-positive treatment uptake levels (Table 3). (One study, by Moussalli et al [17], included an observational phase and an intervention phase that enrolled two separate groups of study participants; for the purpose of calculating treatment uptake levels in this review, the information is regarded as though it represents two studies.) Treatment uptake for groups of hepatitis C RNA-positive study participants ranged from 0% to 61% (median: 30%).

**Table 3:** Treatment uptake as defined by percentage of hepatitis C RNA-positive study participants who received treatment

Author, year	% treated (# treated/# of treatment candidates)	SVR (%) (# with sustained viral response/# treated)
Backmund et al, 2001	50% (50/100)	Intent-to-treat: 36% (18/50)

Broers et al, 2005	61% (11/18)	Intent-to-treat: 56% (6/11) As-treated: 100% (3/3)

Cournot et al, 2004	54% (81/151)	Intent-to-treat: 26% (26/99)^1^

Cullen et al, 2007	10% (3/29)	NA

Ebner et al, 2009	23% (17/75)	Intent-to-treat: 88% (15/17) As-treated: 88% (15/17)

Gazdag et al, 2010	47% (39/83)	NA

Grando-Lemaire et al, 2002	28% (27/97)	Intent-to-treat: 19% (5/27) As-treated: 33% (5/15)

Guadagnino et al, 2007	31% (53/169)	Intent-to-treat: 55% (29/53) As-treated: 85% (29/34)

Hernandez et al, 2009	0% (0/4)	NA

Jack et al, 2009	25% (30/118)	Intent-to-treat: 62% (13/21) As-treated: 77% (13/17)

Jowett et al, 2000	29% (50/172)	Intent-to-treat: 36% (18/50)

Kieran et al, 2011	21% (67/327)	Intent-to-treat: 43% (29/67)

Lindenburg et al, 2011	30% (58/196)	Intent-to-treat: 65% (37/57) As-treated: 77% (37/48)

McCormick et al, 2008	46% (6/13)	Intent-to-treat: 83% (5/6) As-treated: 83% (5/6)

Moussalli et al, 2010^2^	2% (2/113)	NA

Moussalli et al, 2010^2^	38% (85/224)	Intent-to-treat: 43% (37/85)

Perez-Alvarez et al, 2012	56% (15/27)	NA

Perut et al, 2009	9% (12/137)	NA

Reiberger et al, 2011	32% (201/637)	NA

van der Veen, 2009	48% (35/73)	NA

Wilkinson et al, 2008	14% (63/441)	As-treated: 51% (25/49)^3^

Witteck et al, 2011	15% (29/193)	Intent-to-treat: 52% (13/25)

1. Calculated according to the number of treatment outcomes (N=99) rather than the number of patients who initiated therapy (N=81)

2. The study by Moussalli et al includes separate study populations for an observational phase (N=113) and an intervention phase (N=224), and those study populations are represented by two separate records in this table.

3. Calculated according to the number of treatment outcomes (N=49) rather than the number of patients who completed therapy (N=58)

The *treatment uptake level for patients who were HCV RNA-positive and met other criteria* reflects the number of study participants who received treatment as a proportion of the number of study participants who both were RNA-positive for hepatitis C and also met other requirements for treatment, e.g., requirements relating to HCV genotype or to current drug or alcohol intake. Six studies [19,24,32,38-40] reporting on such groups of study participants documented treatment uptake levels ranging from 24% to 76% (median, 55%) (Table 4).

**Table 4:** Treatment uptake as defined by percentage of eligible study participants who received treatment, with eligibility for treatment determined by hepatitis C RNA-positive status and other criteria

Author, year	% treated (# treated/# of treatment candidates)	SVR (%) (# with sustained viral response/# treated)
Defossez et al, 2008	24% (13/55)^1^	NA

Grando-Lemaire et al, 2002	58% (27/47)	Intent-to-treat: 19% (5/27) As-treated: 33% (5/15)

Jack et al, 2009	70% (30/43)	Intent-to-treat: 62% (13/21) As-treated: 77% (13/17)

Jowett et al, 2000	50% (50/100)	Intent-to-treat: 36% (18/50)

Kristensen et al, 2009	33% (6/9)	NA

Lindenburg et al, 2011	76% (58/76)	Intent-to-treat: 65% (37/57) As-treated: 77% (37/48)

1. Study population represents study cohorts from a cross-sectional survey carried out at three time points: 1997 (N=26), 2000 (N=11) and 2003 (N=18).

Data on sustained viral response (SVR) were available for 15 of the 25 studies [16-18,21,22,24,25,31-35,37,38,40] included in the review, with some studies reporting intent-to-treat outcomes, some studies reporting as-treated outcomes, and some studies reporting both (Tables 2, 3, 4). For 14 studies with intent-to-treat data, the proportion of treated study participants attaining a sustained viral response ranged from 19% to 88% (median, 55%). For nine studies with as-treated data, SVR levels ranged from 33% to 100% (median, 80%).

### Treatment uptake levels for key drug-using populations

For 11 studies [20,22-25,27,28,30,33,36,37] included in the review, treatment uptake levels could be calculated specifically for current and former PWID (Table 5). (Other studies had study populations that included non-injecting drug users.) In these studies, the HCV antibody-positive treatment uptake level ranged from 20% to 64% (three studies, median 39%). The HCV RNA-positive treatment uptake level ranged from 0% to 57% (nine studies, median 32%). For two studies reporting treatment uptake in terms of RNA-positive status plus additional criteria, treatment uptake levels were 50% and 71%.

**Table 5:** Treatment uptake in study cohorts of people who inject drugs (current and former)

Author, year	% treated (# treated/# of treatment candidates	SVR (%) (# with sustained viral response/# treated)
Broers et al, 2005	61% of RNA+ (11/18)	Intent-to-treat: 56% (6/11) As-treated: 100% (3/3)

Cournot et al, 2004	39% of antibody+ (58/150)	Intent-to-treat: 22% (15/68)^1^
		
	57% of RNA+ (58/102)	
		
	71% of other (58/82)	

Crespo et al, 2001	64% of antibody+ (268/416)	NA

Gazdag et al, 2010	47% of RNA+ (39/83)	NA

Guadagnino et al, 2007	31% of RNA+ (53/169)	Intent-to-treat: 55% (29/53) As-treated: 85% (29/34)

Hernandez et al, 2009	0% of RNA+ (0/4)	NA

Jowett et al, 2000	20% of antibody+ (50/253)	Intent-to-treat: 36% (18/50)
		
	29% of RNA+ (50/172)	
		
	50% of other (50/100)	

Kieran et al, 2011	21% of RNA+ (67/327)	Intent-to-treat: 43% (29/67)

Perez-Alvarez et al, 2012	56% of RNA+ (15/27)	NA

Reiberger et al, 2011	32% of RNA+ (201/637)	NA

van der Veen, 2009	48% of RNA+ (35/73)	NA

Antibody+ = study participants who are hepatitis C antibody-positive

RNA+ = study participants who are hepatitis C RNA-positive

Other = study participants who are hepatitis C RNA-positive and meet additional treatment criteria

1. Calculated according to the number of treatment outcomes (N=68) rather than the number of patients who initiated therapy (N=58)

Five of the PWID-specific studies reported intent-to-treat SVR levels [22,24,25,33,37]; these ranged from 22% to 56% (median, 43%) (Table 5). Two reported as-treated SVR levels [33,37]; these were 85% and 100%.

For four studies included in the review [18,22,26,35], treatment uptake levels could be calculated specifically for people receiving opioid substitution therapy (OST) (Table 6). (Some other study populations included both people receiving OST and people not receiving OST.) In these studies, the HCV antibody-positive treatment uptake level ranged from 3% to 31% (three studies, median 9%). The HCV RNA-positive treatment uptake level ranged from 10% to 47% (three studies, median 15%). For one study reporting treatment uptake in terms of RNA-positive status plus additional criteria, the treatment uptake level was 72%.

**Table 6:** Treatment uptake in study cohorts receiving opioid substitution therapy

Author, year	% treated (# treated/# of treatment candidates	SVR (%) (# with sustained viral response/# treated)
Cournot et al, 2004	31% of antibody+ (23/75)	Intent-to-treat: 36% (11/31)^1^
		
	47% of RNA+ (23/49)	
		
	72% of other (23/32)	

Cullen et al, 2007	3% of antibody+ (3/104)	NA
		
	10% of RNA+ (3/29)	

Schulte et al, 2010^2^	9% of antibody+ (26/301)	Intent-to-treat: 69% (18/26) As-treated: 86% (18/21)

Witteck et al, 2011^2^	15% of RNA+ (29/193)	Intent-to-treat: 52% (13/25)

Antibody+ = study participants who are hepatitis C antibody-positive

RNA+ = study participants who are hepatitis C RNA-positive

Other = study participants who are hepatitis C RNA-positive and meet additional treatment criteria

1. Calculated according to the number of treatment outcomes (N=31) rather than the number of patients who initiated therapy (N=23)

2. Study cohort received heroin maintenance therapy

Three of the OST-specific studies reported intent-to-treat SVR levels [18,22,35]; these ranged from 36% to 69% (median, 52%) (Table 6). One reported an as-treated SVR level [35]; this was 86%.

Among the 25 studies, only one provided treatment uptake data specifically for current drug users. Cournot et al [22] reported on a study population of current and former PWID, including people receiving OST. Disaggregated data for current drug users indicated an HCV antibody-positive treatment uptake level of 39% (16/41). The HCV RNA-positive treatment uptake level for the same study subset was 50% (16/32). Among current drug users who were HCV RNA-positive and met additional criteria, treatment uptake was 55% (16/29). The intent-to-treat SVR for current drug users in the study by Cournot et al was 16% (data not shown).

### Age and sex of treatment initiators

The mean age of HCV treatment initiators was available for eight studies; it ranged from 29 to 48 (mean, 34) (data not shown). In the nine studies that disaggregated the sex of treatment initiators, the percentage of female treatment initiators ranged from 9% to 85% (mean, 26%) (data not shown).

### Discussion

This systematic review found that hepatitis C treatment uptake is relatively low among drug users in several countries in the WHO European Region, and also that considerable knowledge gaps exist regarding treatment uptake levels in drug users generally and people who inject drugs specifically. There was large variability in treatment uptake levels, with six studies reporting treatment uptake levels below 20% in HCV RNA-positive drug-using study populations, while four reported treatment uptake levels of 50% or higher. However, the median treatment uptake level was alarmingly low, at 30%.

The apparent failure of many European health systems to engage a key population that needs HCV treatment is even more striking in light of the health care resources that appear to be available. For example, the governments of France, Ireland, Spain and the United Kingdom all have reported that the HCV drugs interferon alpha, pegylated interferon, ribavirin, boceprevir and telaprevir are either government-subsidised or are on the national essential medicines lists in those countries [41]. Yet 14 studies from those countries are among the studies demonstrating low treatment uptake among drug users. This may partially reflect recent findings from a survey of civil society organisations, which reports low capacity and extremely limited resources among non-governmental organisations addressing hepatitis [42].

The majority of studies identified by our review included both people who inject drugs and non-injecting drug users in their study populations without providing disaggregated data for these two subgroups. Interestingly, the 11 articles with data specifically for people who inject drugs showed a median treatment uptake level of 32% among HCV RNA-positive PWID – comparable to the median treatment uptake level for the full set of studies. Nonetheless, the fact remains that policy-makers, programme managers and researchers currently possess extremely limited information about the extent to which hepatitis C treatment is reaching the population whose primary risk behaviour – injecting drug use – is the main driver of the hepatitis C epidemic in Europe.

The current situation is likely a legacy of the medical community’s initial outlook on hepatitis C treatment for PWID, which was to not treat current or even past injectors. Although treatment adherence, efficacy and safety have been shown to be quite comparable between PWID and those with no history of drug use, several international and national treatment guidelines in the 1990s and at the beginning of the new millennium excluded PWID from being treated for hepatitis C [8,9,43]. In a revision of its guidelines in 2011, the European Association for the Study of the Liver (EASL) made the important decision to advise instituting antiviral therapy in PWID on stable maintenance substitution treatment after careful individual evaluation by an interdisciplinary team of hepatologists and addiction specialists. As for current drug injectors, an individualised approach after evaluation and close monitoring by an experienced multidisciplinary team were recommended [44].

EASL published a new revision of its guidelines in 2013, and this document reflects the continuing evolution of medical perspectives on the treatment of hepatitis C in drug users and patients on stable maintenance substitution therapy [45]. According to the guidelines, HCV treatment should be considered for PWID on a case-by-case basis, provided they wish to receive it and are willing and able to maintain regular follow-up visits to a multidisciplinary medical service as well as obtain a pre-treatment assessment. The assessment should take into account several factors that may influence adherence to therapy and the likelihood of achieving a sustained viral response, such as ongoing drug use, alcohol consumption, psychiatric disorders, housing, education, employment, and social and financial status. Accordingly, closer monitoring and more intensive multidisciplinary support are needed in certain cases.

The problem is that there appears to be a vast gulf between evidence-based best practice and the decision-making of physicians who encounter HCV RNA-positive drug users seeking care in their clinics. Research indicates that some physicians are reluctant to provide hepatitis C treatment to people who inject drugs, especially those whose drug use is ongoing. For example, a study of private practitioners in Switzerland found that a major reason for non-treatment was intravenous drug use [46].

Even in a research context, where one might hope that treatment criteria for participant-patients are determined in keeping with the established evidence base, current illicit drug use seems to be a legitimate reason for not providing HCV treatment. For example, a 2009 article included in our review described a study that sought “to assess the feasibility and efficacy of antiviral therapy in opioid-dependent patients” by providing one of two HCV treatment regimens to patients at an Austrian addiction clinic. However, participation in the study required meeting one or both of two conditions at the time of enrolment: being in drug maintenance therapy, and abstaining from illicit drugs for at least six months [16].

Other factors commonly associated with injecting drug use may result in the patient’s drug use indirectly creating barriers to HCV treatment. For example, a 2011 article included in the review describes a prospective cohort study in which Dutch drug users were referred to a multidisciplinary hepatitis C treatment unit. Current drug and alcohol use were not considered treatment exclusion criteria. However, in order to be eligible for treatment, drug users “were required to have stable housing and no acute or uncared for juridical or financial impediments” [40]. Another study excluded hepatitis patients coinfected with HIV [31]. While in the context of a study there may be legitimate scientific reasons for excluding certain categories of would-be study participants, in order to avoid potential confounding of the results, these examples do illustrate some of the barriers PWID face in being treated for their life-threatening condition.

Several studies have shown that treatment of PWID with pegylated interferon alpha plus ribavirin has resulted in sustained viral response levels of 54 to 56% [8,9,47] confirming that HCV-infected PWID can be successfully treated. There are some independent factors associated with lower SVR in this group of patients, such as untreated depression, poor socialization and ongoing drug use during HCV treatment [48,49]. On the other hand, PWID in many cases present baseline characteristics shown to be associated with sustained viral response, such as young age and mild liver disease [50]. The mean age in the studies included in this review ranged from 29 to 48, with an aggregated mean of 34.

It has also been reported that a large obstacle to HCV treatment and care for PWID is related to access to care, described as a lack of treatment settings that are suitable for this vulnerable group [12]. Various models for HCV treatment delivery were employed in the articles included in our review. The 2013 EASL guidelines call for a multidisciplinary approach regarding the treatment of HCV infection in PWID including hepatologists and addiction specialists, possibly combining them with other specialists. Therapy can be delivered at community-based clinics, substance use treatment clinics or specialised clinics for the treatment of viral hepatitis. Other options exist as well, such as integrating treatment into primary, secondary or tertiary care, as well as integrating interventions such as directly observed therapy and peer treatment support [5]. The best way to reach the maximum number of infected PWID is to offer the range of various local settings and enable close ongoing collaboration of all involved health professionals [5]. It is now time to further rethink treatment and models of care for PWID.

Treatment not only has the likelihood of curing an individual’s HCV infection, but as in the field of HIV/AIDS, treatment as prevention is increasingly recognised [51]. Mathematical models predict that the transmission rate is reduced when treatment uptake is increased [52-54]. Hence the apparent widespread failure to engage many PWID who have hepatitis C in treatment represents not only missed opportunities to avert potential suffering and death from liver diseases on an individual level, but also missed opportunities to slow an epidemic of major public health significance.

#### Limitations

This literature review has several limitations which may affect the findings. The review primarily contains studies retrieved through PUBMED and EMBASE, which means that the data are subject to publication bias. Drawing on the protocol and methods from a previously published study [15] these problems have been minimised by including missed studies, including studies reported in other languages, provided by a network of drug and infectious disease experts in the European Union. Even with this additional component of the literature search, the 25 articles that met review criteria represent only 12 of the European Region’s 53 Member States. Furthermore, estimates of treatment entry are higher than in that of a recent previous study [15] as there, estimates were only presented for non-intervention (i.e. observational) studies performed in non-clinical settings, whereas in this analysis we included all data available, noting the fact that estimates are partly based on selected populations. The data in our study are thus not directly comparable to the estimates of the previous study, which as its main finding found that overall a lower median (9.5%) of PWID who were diagnosed with HCV among six observational and non-clinical studies had received treatment. Further study including meta-analysis is needed to better understand the biases and generalisability of estimates of treatment access.

Large differences in treatment uptake were observed across the studies included in this review, but since there were major variations in study design and methodology, it is possible that treatment uptake findings may reflect these differences rather than real differences in treatment uptake within and across countries. The following important potential biases may have further influenced the review findings. The included studies were largely undertaken in various types of health care settings, and the drug-using populations served in those settings may not be representative of all people who use drugs. In a number of studies, treatment delivery models were designed with the goal of attracting and retaining people who use drugs, for example by making treatment available at opioid substitution therapy clinics. In light of these considerations, it can be expected that the percentages of people initiating treatment would be higher than the percentage of drug users who are actually initiating treatment in the population at large.

A number of articles were excluded from this review because of methodological shortcomings. Some reported the number of drug users initiating HCV treatment but did not report the total number of drug users who were treatment candidates, making it impossible to determine the proportion of treatment initiators (referred to in this article as the treatment uptake level) [55-59]. Other articles needed to be excluded because they failed to explain how treatment candidates were defined, i.e., it was not clear if patients were considered treatment candidates on the basis of their HCV antibody status, HCV-RNA status or other criteria. Finally, although not part of the exclusion criteria, most studies do not mention whether or not patients were treatment naïve or if previous treatment regimens had been discontinued due to, for example, intolerance/side-effects or failure.

### Conclusions

The availability of hepatitis C treatment data for drug-using populations varies greatly across the WHO European Region. To the extent that treatment uptake levels can be measured, it appears that major differences exist in the proportions of drug users who undergo hepatitis C treatment in different research settings. However, the overall finding of this review suggests that large proportions of people who use drugs, including those who inject drugs, do not enter the treatment pathway and do not receive treatment for their hepatitis C infection.

This observation, coupled with evidence showing that some physicians consider illicit drug use, HIV coinfection and/or related social issues to be counter-indications for hepatitis C treatment, leads us to suggest that a promising strategy for improving the situation is working to increase knowledge and reduce misconceptions among physicians, who play a pivotal role in encouraging or discouraging the initiation of treatment in drug users. There is furthermore great potential to build on integrated health care models, both governmental and nongovernmental, that have demonstrated some success in providing drug users with the medical and social support services that they need in order to optimise their chances of benefitting from hepatitis C treatment. All such efforts must be situated within stronger national efforts to develop HCV treatment policies, strengthen service organisations and ultimately scale up HCV treatment for PWID. In the research realm, future studies should ensure the inclusion of clear criteria to determine if treatment for HCV is needed and should report the indications for starting treatment. This, at a minimum, would include HCV-RNA status, which a number of the excluded articles for this review failed to report.

## Competing interests

The authors declare that they have no competing interests.

## Declarations

This article has been published as part of *BMC Infectious Diseases* Volume 14 Supplement 6, 2014: Viral Hepatitis in Europe. The full contents of the supplement are available online at http://www.biomedcentral.com/bmcinfectdis/supplements/14/S6. The publication charges for this supplement were funded by AbbVie as an unrestricted grant to Rigshospitalet, the University of Copenhagen. AbbVie further funded the printing of the supplement with additional financial support from the Drug Prevention and Information Programme (DPIP) of the European Union.
